# Retraction Note: Chronic neuroinflammation and cognitive impairment following transient global cerebral ischemia: role of fractalkine/CX3CR1 signaling

**DOI:** 10.1186/s12974-015-0442-1

**Published:** 2015-11-26

**Authors:** Teresita L. Briones, Julie Woods, Magdalena Wadowska

**Affiliations:** Department of Adult Health, Wayne State University, 5557 Cass Ave.Cohn Bldg, Rm 344, Detroit, MI 48202 USA; Department of Biobehavioral Health Science, University of Illinois at Chicago, Chicago, IL 60612 USA

This article [1] has been retracted by the Editors-in-Chief. Based on the report of an inquiry conducted by Wayne State University and additional analysis conducted by the Office of Research Integrity (ORI) [2], the ORI informed the journal that the first author Dr Teresita L. Briones engaged in research misconduct by falsifying and/or fabricating data by falsely reporting the results of western blot experiments and that data reported in Figures 2A and 2C in [1] were duplicated, reused and falsely relabelled, and claimed to represent different experiments. We have not been able to contact any of the authors.
